# Effects of an Hourly Bolus Postruminal Infusion of Flaxseed Oil or Palm Oil on Circulating Fatty Acid Concentrations and Hepatic Expression of Pyruvate Carboxylase and Phosphoenolpyruvate Carboxykinase in Dairy Cattle

**DOI:** 10.3390/ani13223572

**Published:** 2023-11-19

**Authors:** Linda M. Beckett, Victor M. R. Malacco, Susan Hilger, Theresa M. Casey, Shawn S. Donkin

**Affiliations:** 1 Department of Animal Sciences, Purdue University, West Lafayette, IN 47907, USA; 2 College of Agriculture, Oregon State University, Corvallis, OR 97331, USA

**Keywords:** dairy cattle, flaxseed oil, palm oil

## Abstract

**Simple Summary:**

This study discusses the impacts of postruminally infusing palm oil or flaxseed oil in conjunction with propionate or acetate on blood metabolites and hepatic pyruvate carboxylase (*PC*) and phosphoenolpyruvate carboxykinase (*PCK1*) expression. The flaxseed infusion changed plasma C18:3n-3 *cis* but did not affect *PC* or *PCK1* expression. The palm oil infusion did not affect plasma metabolites or *PC* or *PCK1* expression.

**Abstract:**

Palmitic (C16:0), α-linolenic acid (C18:3n-3 *cis*), and propionate regulate bovine pyruvate carboxylase (*PC*) and phosphoenolpyruvate carboxykinase (*PCK1*) expression in vitro. The objective of this experiment was to determine the impact of C16:0, C18:3n-3 *cis*, propionate, and acetate postruminal infusions on hepatic *PC* and *PCK1* expression. We hypothesized that circulating fatty acids alter hepatic *PC* and *PCK1* in lactating dairy cows. Acetate, propionate, palm oil, and flaxseed oil were supplied postruminally to lactating cows (*n* = 4) using two 4 × 4 Latin square studies. For Experiment 1, cows were infused on an hourly basis with either a bolus of propionate, acetate, or the combination of propionate and palm oil, or acetate and palm oil, and Experiment 2 was similar, but flaxseed oil replaced palm oil. Flaxseed infusions increased plasma concentration and the molar percent of C18:3n-3 *cis* and decreased C16:0 but did not affect *PC* or *PCK1* expression. Palm infusions did not affect blood metabolites or the hepatic expression of *PC* or *PCK1*. The lack of responses to short-chain fatty acid infusions and changes in circulating long-chain fatty acids in mature cattle are not suitable models to study the effects of α-linolenic acid and propionate on bovine *PC* and *PCK1* expression previously observed in vitro.

## 1. Introduction

During peak lactation, nearly 3 kg of glucose is removed from circulation by the mammary gland each day to support milk synthesis in high-producing dairy cows [1]. As a ruminant, dairy cattle must synthesize 90% of the glucose required for milk synthesis [2,3] with approximately 80% produced by hepatic gluconeogenic capacity [4,5]. Gluconeogenesis is regulated by substrate availability, the activity of the tricarboxylic acid (TCA) cycle and gluconeogenic enzymes, and the flux of carbons toward glucose [4,6]. The main substrates used for gluconeogenesis in dairy cattle are propionate, lactate, glycerol, and amino acids [4]. Propionate and lactate are rumen-derived, and propionate contributes 60 to 74% to glucose production and lactate contributes approximately 20% [4]. To be utilized for gluconeogenesis, these substrates are first converted to TCA cycle intermediates and are, in turn, drawn out of the cycle through phosphoenolpyruvate for glucose synthesis. The TCA cycle intermediate, oxaloacetate (OAA), is the substrate in the first committed step of gluconeogenesis, which converts OAA to phosphoenolpyruvate (PEP), ultimately drawing OAA carbons from the TCA cycle. 

The pool size of OAA is determined primarily by the activity of two enzymes, pyruvate carboxylase (PC) and phosphoenolpyruvate carboxykinase (PEPCK) [6], but other enzymes can moderate OAA pool size, like malate dehydrogenase. PC mediates the primary anaplerotic reaction that produces OAA by catalyzing the carboxylation of pyruvate. PEPCK is a cataplerotic enzyme and pulls OAA from the TCA cycle by irreversibly converting it to phosphoenolpyruvate, which is then used for gluconeogenesis. Thus, PC replenishes and provides OAA, whereas PEPCK pulls OAA toward gluconeogenesis. PEPCK is encoded by two genes, *PCK1* and *PCK2*, which encode the cytosolic and mitochondrial isoforms, respectively [7,8]. The expression of *PCK1* is regulated by glucocorticoids in bovine hepatocytes in vitro [9] and in vivo in cattle [10] and goats [11]. Glucagon increases gluconeogenesis in primary bovine hepatocytes [12] and stimulates the expression of *PCK1* in ruminants in vivo [13,14]. Both hepatic *PC* and *PCK1* are responsive to physiological changes at calving [15,16] and propionate supply [17]. 

The activity of the PC enzyme in synthesizing OAA and the activity of PEPCK in pulling OAA carbon from the TCA cycle are central to the regulation of hepatic gluconeogenesis and carbon flux to glucose or TCA cycle oxidation. Intravenous infusions of propionate, in pre-ruminating calves, increased hepatic *PCK1* expression, but mRNA levels of *PC* were not affected [17], whereas the treatment of primary cultures of calf hepatocytes with propionate increased the expression of *PC* and *PCK1* [9]. Analysis of the bovine *PCK1* promoter activity indicates that propionate induces *PCK1* promoter activity [18]. The combination of these approaches indicates that propionate modulates *PC* and *PCK1* expression levels in vitro and in vivo. 

Our in vitro studies also support the idea that long-chain fatty acids modulate *PC* expression, which may affect the flow of carbons toward gluconeogenesis. The expression of bovine *PC* mRNA in Madin–Darby Bovine Kidney (MDBK) cells decreased with palmitate exposure [19], whereas the treatment of cells with an equal ratio of palmitate (C16:0) to α-linolenic acid (C18:3n-3 *cis*) recovered *PC* expression levels to the baseline [19]. These data are supported by greater responsiveness of the bovine *PC* proximal promoter to α-linolenic acid treatment relative to palmitic acid [20]. The induction of *PC* expression was greatest when both palmitate and α-linolenic acid were present in the culture. In silico analysis of the nucleotide sequence in the *PC* proximal promoter region found binding sites for peroxisome proliferator-activated receptor α and sterol regulatory element binding protein [20], supporting the potential for fatty acid regulation of *PC* expression through induction of these transcription factors. 

During lactation, there are dynamic shifts in the circulating levels of short-chain and long-chain fatty acids as a consequence of changes in intake, rumen fermentation, energy demands for milk production, mobilization status of adipose tissue as a source of long-chain fatty acids, and changes in diet composition that affect short-chain fatty acid (SCFA) production. Given that previous findings support a role for palmitate, α-linolenic acid, and propionate in the regulation of the hepatic expression of *PC* and *PCK1* [19], we postulated that shifts in postruminal supply of short- and long-chain fatty acids leads to alterations in gluconeogenic capacity in part through changes in the relative expression of *PC* and *PCK1* mRNA. Our working hypothesis is that gluconeogenesis in lactating dairy cattle is the net result of the relative activity of PC to anaplerotically ‘push’ carbon toward OAA and the activity of PEPCK to cataplerotically ‘pull’ carbon from the OAA pool. The objective of this experiment was to determine if postruminally supplied propionate, palmitate (in the form of palm oil), and α-linolenic acid (in the form of flaxseed oil) would affect *PC* and *PCK1* expression and their relative ratios in lactating dairy cows. Because in vitro studies found palmitate decreased the expression of *PC* and α-linolenic acid rescued this response, we hypothesized that the ratio of *PC* to *PCK1* would be lower when cows were infused with palm oil combined with acetate and be similar when cows were provided flaxseed oil. Additionally, we hypothesized that *PC* expression would be marginally reduced when palm oil is supplied with propionate because propionate induces both *PC* and *PCK1* despite the potential negative effect of palm oil on *PC* mRNA. 

## 2. Materials and Methods

### 2.1. Animal Care and Handling

Animal care and handling protocols were reviewed and approved by the Purdue University Animal Care and Use Committee prior to beginning the experiment at the Purdue Animal Science Research and Education Center Dairy Unit (Protocol # 1906001913). A power analysis was conducted for Latin square design using the protocol by [21] that calculates power for Latin squares using the PROC MIXED procedure in SAS 9.4, and variations in circulating C16:0 and C18:3n-3 *cis* were evaluated. The power of the separate squares was above 0.80 with a 5% significance level. The reported critical F-value was 3.86, with an F-value greater than 1 typically meaning there are different population means. Additionally, other studies that postruminally infused flaxseed (or linseed) oil have utilized Latin square designs, specifically 3 × 3 [22] and 5 × 5 [23], and another study that evaluated the impact of propionate infusion on hepatic *PC* and *PCK1* expression utilized four animals per treatment and successfully elicited a response of propionate on hepatic *PCK1* expression [17]. Four early-lactation Holstein cows fitted with rumen cannulas during the previous lactation were used in two separate 4 × 4 Latin square design studies. Cows had an average (± standard deviation) of 60.8 ± 11.6 days in milk (DIM), DIM ranged from 45 to 74 d at the start of the experiments, and cows weighed 599 ± 50 kg (average ± standard deviation). 

Cows were housed in tie-stalls and released at 0500 and 1600 for milking. Average daily milk production at the beginning of Experiment 1 was 41.4 ± 4.4 kg. Cows were fed a total mixed ration once daily at 0600 in amounts that resulted in 10% feed weight back over a 24 h period and had free access to water. Periods were 7 d in length with the 1 d consisting of an hourly bolus postruminal infusion and the following 6 d serving to washout treatments. An hourly bolus postruminal infusion was selected because preliminary tests prior to the experiment initiation indicated poor efficacy of delivering the flax and palm oils using a continuous infusion. Postruminal infusion lines were prepared following [24] and were placed through the reticulo-omasal orifice into the omasum 12 h prior to the start of infusion. The correct placement of infusion lines was verified, at a location distal to the reticulo-omasal orifice, 15 min prior to the start of the first infusion hour and at the conclusion of the infusion period. 

### 2.2. Treatments

Postruminal infusions consisted of (a) 434 g food grade (purity > 95%) sodium acetate in 5.2 L of distilled water (Ac; Sigma Aldrich, St. Louis, MO, USA), (b) 202 g food grade (purity > 95%) sodium propionate (Pr; Sigma Aldrich, St. Louis, MO, USA) in 5.2 L of distilled water, (c) 87 g emulsified palm oil (Greener Life Club, Sebring, Florida) in combination with 434 g of sodium acetate in 5.2 L of distilled water (Palm-Ac), or (d) 87 g of emulsified palm oil with 202 g of sodium propionate in 5.2 L of distilled water (Palm-Pr). The quantities of acetate and propionate were calculated to be isoenergetic across the treatments with the palm oil product set at 87 g. Given the amount of acetate and propionate provided in the infusion, triglyceride product dosage was calculated lower than previously reported dosages [25,26] and palm oil dose was calculated to be less than flaxseed oil dose due to the level of saturation contributing to greater energy density. Triglyceride products were chosen because of the potential applicability of these products in dairy cattle diets since sources of fatty acids are typically oils, which are a blend of fatty acids. In addition, abomasal infusions of fatty acids or triglycerides at the rate of 250 g/d decreased dry matter intake, but triglyceride products had a 2-fold less impact on dry matter intake [26]; thus, triglyceride products were chosen. The palm oil (87 g) was liquified by heating the triglyceride product to 70 °C and was then emulsified with the addition of 5.3 g of Tween 40 (Sigma Aldrich, St. Louis, MO, USA) to achieve 1% of the total infusate volume. The palm oil and Tween 40 were mixed using a conventional blender and added to either the sodium acetate or propionate solutions previously warmed to 70 °C. Tween 40 (5.3 g) was added to all treatments regardless of whether the treatment contained a triglyceride product. Infusions were administered as a 660 mL bolus dose every h for 8 h. The total fluid volume infused over 8 h for each treatment was 5.28 L. The infusion time length was chosen based on a previous study by [17] who elicited changes in circulating propionate concentrations using an 8 h infusion for one day. Additionally, pulse dosing of fats has been used by [23,27] although the pulsing dosing occurred over a longer treatment period than the present study. 

Experiment 2 consisted of a postruminal infusion of either (a) 434 g of sodium acetate in 5.2 L of distilled water (Ac 2), (b) 202 g of sodium propionate in 5.2 L of distilled water (Pr 2), (c) 151 g of emulsified flaxseed oil (Zatural, Eden, Idaho) with 434 g of sodium acetate in 5.2 L of distilled water (Flax-Ac), or (d) 151 g of emulsified flaxseed oil with 202 g of sodium propionate in 5.2 L of distilled water (Flax-Pr). Flaxseed oil (151 g) was heated to 70 °C and Tween 40 (5.3 g) was added to total 1% of infusate volume. Tween 40 was added to all treatments. The flaxseed oil and Tween 40 were mixed using a conventional blender and added to either the sodium acetate or propionate solutions previously warmed to 70 °C. Infusions were administered as a 660 mL dose every h for 8 h, and the total infusate volume for each treatment was 5.28 L. 

### 2.3. Diet and Feed Analysis

The lactating cow diet is shown in Table 1. Fresh feed samples were collected once per 7 d period. Feed samples were analyzed for dry matter (DM) by drying as-fed samples at 55 °C in an air force oven for 24 h. Neutral detergent fiber (NDF), acid detergent fiber (ADF), starch, fat, and ash were analyzed at DairyOne (Ithaca, NY, USA). 

### 2.4. Sample Collection

Indwelling catheters were placed in both jugular veins approximately 30 min prior to the first blood sample. A total of 10 blood samples were collected over the 8 h infusion period, with two samples collected at −15 min and −5 min relative to the first infusion dose and the remaining samples collected every h following the initiation of postruminal dosing. Consequently, the last blood sample was taken 1 h after the last treatment dosing. Three blood samples were collected each hour; 10 mL collected in a NaF blood collection tube (BD Vacutainer Glass Blood Collection Tubes with Fluoride, Fisher Scientific, Waltham, MA, USA), 10 mL in a serum separator tube (BD Vacutainer Venous Blood Collection Tubes, Fisher Scientific), and 10 mL in a sodium heparin tube (BD Vacutainer Glass Blood Collection Tubes with Sodium Heparin, Fisher Scientific). Samples containing NaF and heparin were immediately placed on ice, transported to the lab, and centrifuged at 1500× *g* min for 15 min. Serum was permitted to clot at room temperature for 30 min prior to being centrifuged at 1500× *g* min for 15 min. Serum and plasma were removed and stored at −20 °C until plasma glucose, non-esterified fatty acid (NEFA), and plasma fatty acid profile analysis. 

### 2.5. Liver Biopsy Sampling

Immediately following the final blood sampling, cows were immediately moved from tie-stalls to a working chute for liver biopsy. Liver samples were collected by blind percutaneous needle biopsy described in [15]. Briefly, the intercostal space between the 11th and 12th rib was shaved and scrubbed with betadine and swabbed with 70% ethanol. Ten milliliters of lidocaine were injected subcutaneously overtop the intercostal space. After numbness was confirmed, a 1 cm incision was made in the 11th and 12th intercostal space using a scalpel blade. A 9 mm custom biopsy needle was passed through the peritoneum and to the liver. The stylus was retracted, and the instrument was inserted 2 to 3 cm into the liver. A vacuum created inside the stylus allowed for the extraction of liver tissue into the biopsy needle. Approximately 250 mg of liver tissue was collected. The tissue was placed in 5 mL of Trizol reagent, immediately snap-frozen in liquid nitrogen, and stored at −80 °C until further analysis. The incision site was sutured using a non-absorbable #2 suture. Cows were monitored once daily for seven days after the biopsy, and on d 7 sutures were removed. 

### 2.6. Fatty Acid Analysis

Palm oil and flaxseed oil were analyzed for their fatty acid content. Samples were prepared using the AOCS Official method Ce 2-66 with the alternative method for fats and oils. Step 4 was employed [28] to form methyl esters of fatty acids. In short, 200 μL of oil in 2 mL of hexane was combined with 100 μL of 2 N KOH in methanol and vortexed. The mixture was let to rest for five minutes at room temperature. Then, 3 mL of 6% (*w*/*v*) potassium carbonate was added and mixed, and the mixture was centrifuged at 1000 rpm for 5 min. The upper hexane layer was transferred to a gas chromatography vial. The gas chromatography settings and parameters were as previously described [29,30].

### 2.7. Plasma Fatty Acid Analysis

The plasma sample collected after the last infusion dose was analyzed for the fatty acid profile using liquid chromatography–mass spectrometry (LC/MS). Heptadecanoic acid (C17:0) was used as an internal standard [31,32] and added to the blood sample prior to fatty acid extraction. Heptadecanoic acid was chosen because it is lowly abundant in circulation at approximately 0.4 g/100 g [33], and in milk, C17:0 ranges from 0.6% to 0.7% across colostrum, transition, and mature milk [34]. A 40 μL aliquot of plasma was added to 2 mL of 30% heptane in isopropanol (*v*/*v*) and 1 mL of 0.003 M sulfuric acid. The organic layer was removed and dried under nitrogen gas. The sample was re-constituted in 90% acetonitrile with 0.5 mM ammonium acetate. The LC/MS was run using a 1260 Infinity II LC, Zorbax Eclipse Plus C18 column, and a 6160A single-quadrupole mass spectrometer with electrospray ionization (Agilent Technologies, Santa Clara, CA, USA). Mobile phase A was 80% acetonitrile with 0.5 mM ammonium acetate, and mobile phase B was 100% acetonitrile with 0.5 mM ammonium acetate. The flow rate was 0.4 mL/min. Fatty acids were eluted isocratically with 45% mobile phase A and 55% mobile phase B followed by a column wash. Free fatty acids were detected in negative mode using selective ion monitoring; compounds were identified using the retention time and mass-to-charge ratio and quantitated using external standard curves for each fatty acid.

### 2.8. Plasma Glucose and Non-Esterified Fatty Acid (NEFA) Analysis

Plasma glucose was analyzed using a blood glucose kit (FUJIFILM Wako Diagnostics, Mountain View, CA, USA). Non-esterified fatty acids (NEFAs) were analyzed using a blood NEFA kit (FUJIFILM Wako Diagnostics, Mountain View, CA, USA). Absorbance values for glucose were measured at 505 nm and, NEFAs were measured at 550 nm using a Tecan Spark 10M multimode plate reader (Tecan, Zürich, Switzerland). Each sample was analyzed in triplicate, and raw absorbance values were averaged. 

### 2.9. RNA Extraction, Isolation, and RT-qPCR

Liver samples in Trizol were homogenized for 30 s using an IKA Works Inc. (Wilmington, NC, USA) homogenizer. The homogenizer probe was rinsed with 5 mL of nanopure water, 5 mL of 70% ethanol, and 5 mL of fresh Trizol between samples. After homogenization, samples were incubated at RT for 5 min for the complete dissociation of the nucleoprotein complex; then, 200 µL of chloroform per 1 mL of Trizol was added, and the samples were mixed by inversion. Samples were incubated on ice for 3 min and then centrifuged at 12,000× *g* for 15 min at 4 °C. The aqueous phase was removed from the sample and transferred to a new tube. Five hundred µL of isopropanol per 1 mL of Trizol was added to an aqueous extract, incubated on ice for 10 min, and then centrifuged at 12,000× *g* for 10 min at 4 °C to pellet RNA. The supernatant was discarded, and the pellet was resuspended in 75% ethanol per 1 mL of the original Trizol volume and centrifuged at 7500× *g* for 5 min. Ethanol was removed, and the pellet was resuspended in 200 µL of RNase-free water. RNA was cleaned and DNAse treated using a Qiagen RNeasy Mini Kit (Qiagen, Hilden, Germany) following the manufacturer’s protocol. Samples were stored at −80 °C until further analysis. 

RNA was quantified using a nanodrop spectrophotometer ND-1000 (Thermo Fisher Scientific, Waltham, MA, USA). An aliquot of RNA was size-separated by agarose gel electrophoresis ([15]. The gel was imaged using a UVDI gel electrophoresis imaging box (Major Science, Saratoga, CA, USA). RNA quality was assessed from the ratio of 28S to 18S ribosomal RNA. Single-stranded complementary DNA (cDNA) was synthesized from 2 µg of RNA for each sample using the Qiagen Omniscript RT kit (Qiagen, Hilden, Germany) following the manufacturer’s protocol. Random decamers, oligo DT, dNTP, 10× Buffer RT, RNase Inhibitor, and RTO were combined into a microcentrifuge tube along with the cDNA. The tubes were placed in a heat block at 37 °C for 1 h.

A real-time quantitative polymerase chain reaction (RT-qPCR) was used to measure relative gene expression. cDNA from each sample was combined with 23 µL of Master Mix that contained Brilliant III Ultra-Fast SYBR Green QPCR Master Mix (Agilent Technologies, Santa Clara, CA, USA), a 1:500 dilution of ROX, RNase/DNase/Nuclease-free water, the forward primer (2.5 pmol/µL), and the reverse primer (2.5 pmol/µL) of the gene of interest. The final concentration of the primers was 100 nM. All RT-qPCR samples were analyzed on a Stratagene Mx 3005p^TM^ (Agilent Technologies, Santa Clara, CA, USA). Both GAPDH (NM_001034034.2 FOR: CATGTTTGTGATGGGCGTGAACCA; REV: TGATGGCGTGGACAGTGGTCATAA) and 18S (NR_036642.1 FOR: ACCCATTCGAACGTCTGCCCTATT; REV: TCCTTGGATGTGGTAGCCGTTTCT) were evaluated as reference genes. The reaction quality of the reference genes was assessed using an efficiency of 90 to 110%. The test genes included PC (NM_177946.4 FOR: CCACGAGTTCTCCAACACCT; REV: TTCTCCTCCAGCTCCTCGTA) and PCK1 (NM_174737.2 FOR: AACCTGGCCATGATGAACCCTACT; REV: ACTCCTTGCCCTTCCAGGAAATGA). The cDNA sample ensured that all samples fell on the standard curve and achieved an efficiency between 90 and 110%. Relative gene expression was calculated following the protocol by [19]. 

### 2.10. Statistical Analysis

Data were analyzed using the MIXED procedure in SAS 9.4 (SAS, Cary, NC, USA) [35]. The following model was used for all data:

Y_ijk_ = μ + a_i_ + b_j_ + c_k_ + e_ijk_ (1)

where Y_ijk_ is the response variable, u is the overall mean, a_i_ is the fixed effect of the ith treatment, b_j_ is the fixed effect of the jth period, c_k_ is the random effect of the kth cow, and e_ijk_ is the residual. For the plasma glucose and NEFA concentration models, the fixed effect and repeated measure of time were added, and Tukey’s honest significant difference post hoc test was used to identify specific time effects. The covariance structure was autoregressive, which was determined by the lowest Akaike’s Information Criterium (AIC). Orthogonal contrasts were employed to compare the effect of triglyceride product (palm or flaxseed oil), SCFAs (acetate compared to propionate), and the interaction (palm oil × SCFA or flaxseed oil × SCFA) for each experiment. Each experiment was analyzed separately, and means were not compared across squares. Means were determined to be different when *p* < 0.05 and tended to be different when 0.05 ≤ *p* ≤ 0.10. 

## 3. Results

### 3.1. Effect of Postruminal Infusion of Palm Oil and Flaxseed Oil on Plasma Fatty Acid Profile, Glucose, and Non-Esterified Fatty Acid (NEFA) Levels

Analysis of the lipid sources indicated greater C16:0 and C18:1n-9 *cis* in palm oil and greater C18:3n-3 *cis* in flax oil (Table 2). There were no significant overall treatment effects (*p* > 0.05), in either experiment, on milk production collected on the day of the infusion (Table 3 and Table 4). 

Postruminal infusion of palm oil had no effect (*p* > 0.10) on plasma glucose or plasma NEFA (Experiment 1; Table 3), and there was no significant effect (*p* > 0.10) of palm oil infusion on the profile of plasma fatty acids. There was no significant time effect on plasma glucose or NEFA concentration (*p* > 0.10). There was, however, a tendency (0.05 ≤ *p* ≤ 0.10) for acetate infusions to increase C20:5 molar proportion (Experiment 1, Table 3) and for propionate infusion to increase blood C18:0 concentration (Experiment 2, Table 4). 

Postruminal infusion of flaxseed oil significantly (*p* < 0.05) increased plasma C18:3n-3 *cis* concentration relative to the infusion of SCFAs (acetate or propionate) alone (Table 4), and a plasma concentration of C18:0 tended to decrease (*p* = 0.07) with flaxseed infusion. Flaxseed infusion decreased (*p* < 0.05) plasma C16:0 and C14:0 molar proportion and tended to decrease C14:1 molar proportion in plasma (0.05 ≤ *p* ≤ 0.10). There was also a tendency (0.05 ≤ *p* ≤ 0.10) for flaxseed to increase NEFA concentration (Table 4). There was a significant time effect (*p* < 0.05) on plasma glucose concentration, with the baseline timepoint being different than the sample at hours 4 and 7, but the 5, 6, and 8 h timepoints did not differ from the baseline. There was a significant time effect (*p* < 0.05) on plasma NEFA concentration, with hour 1 being different than all the other infusion timepoints (hours 2–8), but not the baseline timepoint. The baseline plasma glucose and NEFA were both similar prior to the start of the infusions for both experiments. 

### 3.2. Effect of Postruminal Infusion of Palm Oil and Flaxseed Oil on Hepatic PC and PCK1 Expression

The hepatic expression of *PC* and *PCK1* and the ratio of *PC* to *PCK1* were not significantly altered by palm oil infusion (Table 5). Likewise, flax infusion had no effect on *PC* and *PCK1* or the ratio of *PC* to *PCK1*. 

## 4. Discussion

The enzymes, PC and PEPCK, play key roles in the liver by directing the flow of carbons in and out of the TCA cycle and provide key substrates for gluconeogenesis and the capacity for acetyl-CoA oxidation. In vitro studies from our lab support a potential role for SCFA and NEFA in regulating the expression of *PC* and *PCK1* [9,19]. The objective of the experiments described here was to determine changes in hepatic *PC* and *PCK1* in response to the postruminal infusions of palm oil or flaxseed oil in combination with SCFA. Because C16:0 is a potent repressor of *PC* expression in vitro, we predicted that palm oil, which contains 45% palmitate, would decrease hepatic *PC* mRNA abundance in response to increased circulating levels of C16:0. However, the infusion of palm oil in Experiment 1 failed to alter the concentration or molar percent of circulating C16:0. Likewise, because in vitro studies indicate that C18:3n-3 *cis* counteracts the effects of palmitic acid to depress *PC* mRNA expression, we reasoned that the direct postruminal infusion of flax oil, containing 58.0% C18:3n-3 *cis*, would increase *PC* expression given the high level of circulating C16:0 in dairy cattle. 

The lack of response of the plasma fatty acid profile to palm oil in these experiments may have been the result of the inclusion of Palmit 80 (Global Agri-trade Corporation, Rancho Dominguez, CA) in the basal diet a dry, a beaded ingredient consisting of 85% palmitic acid (C16:0). Based on the average DM intake prior to the infusion interventions of 24.6 kg/d, cows with a 0.90% inclusion rate were already receiving 0.188 kg (24.6 kg/d × 0.85 palmitate × 0.009 inclusion rate) of palmitate daily. The infusion protocol of 87 g palm oil, containing 45% palmitate, increased the estimated daily palmitate supply by 39 g over the 8 h of infusion or approximately 117.45 g/24 h. Palmitic acid, continuously infused into the abomasum of dairy cows [36] at a rate of 295 g/d in cows fed base diets containing 0.28% Palmit 80, slightly increased plasma C16:0 by 12 to 17% compared with the infusion of lipid sources low in palmitate (behenic acid and algae oil). Given the level of palmitate infused and the inclusion of palmitate in the base diet, the lack of change in circulating palmitate is not surprising in our experiment. The circulating palmitic acid pool is highly abundant in early lactation dairy cows [37]; therefore, in order to significantly change the circulating palmitic acid pool, our infusion dosage would likely have needed to be larger, last for a longer duration, or be used with a base diet devoid of palmitate, or a combination of all conditions.

Flaxseed infusion increased the concentration of plasma C18:3n-3 *cis* almost eight times in both the Flax-Pr and Flax-Ac treatments compared to Pr 2 and Ac 2. This identified that an 8 h infusion was adequate to appropriately enrich circulating fatty acids from a triglyceride product. This was an expected result because lactating dairy cattle supplemented with dietary flaxseed oil had a 3.4 times greater concentration of plasma α-linolenic acid compared to their controls [38]. In addition, when lactating dairy cows were fed micronized or extruded flaxseed oil plasma concentrations, saturated fatty acids decreased in response to additional C18:3n-3 *cis* [39]. Despite these expected responses, we failed to observe changes in hepatic *PC* or *PCK1* expression. 

The lack of effects of SCFA alone or in combination with either palm oil or flaxseed oil on hepatic *PC* and *PCK1* expression, despite significant changes in circulating fatty acid profiles, suggest either a lack of response in vivo to shifts in plasma fatty acid concentration or an inability to induce or detect a response under these experimental conditions, or a combination of these possibilities. The hypothesis for these experiments was based on combinations of previous work using bovine hepatocytes, postruminal infusion of propionate [17], and research using reporter genes in hepatoma cells [19,20] that point to the regulation of *PC* and *PCK1* by propionate, palmitic, and linolenic acids. In these instances, the infusion of propionate was continuous, and exposure of hepatocytes to NEFAs was for at least 21 h. Furthermore, the exposure of cells to propionate in previous cell experiments was at a high physiological concentration relative to in vivo portal blood concentrations [40] and in a system devoid of homeorhetic responses in circulating hormones and nutrients that are typical in vivo. Lastly, acetate and propionate are rapidly cleared from circulation once absorbed to be used for milk fat synthesis and gluconeogenesis, respectively [4,37]. Therefore, the lack of response to hepatic gene expression is likely due to the frequency and length of infusion. 

The current experiment utilized an in vivo model, where SCFA and triglyceride products were bolus-dosed hourly over a period of 8 h. Previously, we showed that the infusion of propionate, compared with glucose infusion, maintained *PCK1* levels in the bovine liver but was not different from the infusion of water [17], and the effects of propionate were through counteracting the effects of elevated insulin concentrations. Although plasma insulin was not measured in the current experiments, the infusion of acetate to match the energy supply would not be expected to result in elevated plasma insulin [41]. Therefore, it is not surprising that there is a lack of effect of SCFA treatment on *PCK1* expression. 

The current study provides evidence that the postruminal bolus dosing of lipid sources is a useful experimental model to alter the short-term circulating supply of linolenic acid in lactating dairy cows. The same may be true for other fatty acids that are uniquely elevated in lipid sources, depending on their profile relative to dietary fatty acid sources, endogenously produced fatty acids, and circulating profiles. Despite conducting a power analysis that supported a 4 × 4 Latin square design for these experiments, caution should be taken when interpreting these data due to the limited sample size and some treatment difference tendencies. In addition, at the start of the experiment, the DIM range encompassed pre-peak and post-peak milk production, despite all animals being in early lactation; therefore, further caution should be used when interpreting these data. Additional effort is needed to determine the impact of the duration of the change in circulating fatty acids on hepatic gene expression and other metabolic parameters. 

## 5. Conclusions

Postruminal infusion of palm oil product, which is 45% palmitate, in early lactation had no effect on the circulating levels of fatty acids measured after infusion conclusion and no effect on hepatic expression levels of *PC* and *PCK1*. When flaxseed oil, which is 58% α-linolenic acid, is infused into the abomasum of mid-lactation cows, the plasma C18:3n-3 *cis* molar proportion increased, whereas the molar proportion of C16:0 decreased. However, there was no effect on hepatic *PC* and *PCK1* expression. The data provided in this paper provide insight into conducting postruminal infusions of triglyceride products. Further studies are necessary to determine if altering the postruminal availability of fatty acids can alter *PC* and *PCK1* hepatic expression to affect gluconeogenic capacity.

## Figures and Tables

**Table 1:** Diet ingredients and nutrient composition presented as averages or averages ± standard deviation ^1^.

Ingredients	% Inclusion
Corn Silage	49.8
Alfalfa Haylage	14.0
Wheat Straw	3.20
Alfalfa Hay	5.30
High Moisture Shelled Corn	9.70
Corn Grain, Finely Ground	2.50
Soybean Meal	3.00
Lactating Cow Supplement ^2^	5.60
AA Blend ^3^	3.20
QLF TMR Blend ^4^	2.80
Palmit 80 ^5^	0.90
**Nutrient Composition**	**% DM ^6^**
Dry Matter	50.2 ± 1.40
Crude Protein	15.5 ± 0.51
Neutral Detergent Fiber	27.5 ± 2.14
Acid Detergent Fiber	17.8 ± 1.84
Crude Fat	5.10 ± 0.29
Ash	8.40 ± 0.33
Net Energy Lactation, Mcal/kg	0.36 ± 0.01

^1^ Dry matter basis unless indicated. ^2^ Lactating cow supplement mixed at Purdue University; ingredients lists as % DM basis: finely ground corn grain 2.68%; calcium carbonate 0.62%; soybean meal 0.47%; sodium biocarbonate 0.47%; salt 0.24%; calcium phosphate mono 0.19%; DCAD Plus 0.19%; Omnigen AF (Phibro Animal Health; Teaneck, NJ, USA) 0.12%; Diamond V XP (Diamond V, Cedar Rapids, IA, USA) 0.11%; magnesium oxide 0.12%; calcium sulfate 0.11%; Yellow Grease 0.10%; Vitamin and Mineral premix 0.09%; Vitamin E 0.01%; Rumensin 0.004%. ^3^ AA Blend contains Soy Plus 2.7% of DM, Blood Meal 0.38% of DM, Smartamine M 0.03% of DM, Select BioCycle Plus Concentrate 0.03% of DM, AjiPro L 0.01% of DM. ^4^ QLF TMR Blend = Quality Liquid Feed (QLF, Dodgeville, WI, USA)) TMR supplement for lactating dairy cows; 63% dry matter; 2.4% NFC on DM basis; 11.1% ash on a DM basis. ^5^ Palmit 80 = palmitic acid supplement from Global Agri-trade Corporation, Rancho Dominguez, CA, USA). ^6^ Percent of dry matter.

**Table 2:** Fatty acid profile for palm and flaxseed oil used for 8 h postruminal infusion. Data are presented as averages ± standard deviation.

Fatty Acid	Palm Oil, % mol	Flaxseed Oil, % mol
14:0	1.10 ± 0.01	0.07 ± 0.04
14:1	ND	ND
15:0	0.05 ± 0.00	ND
16:0	45.0 ± 0.23	5.14 ± 0.04
16:1	0.16 ± 0.00	0.07 ± 0.00
17:0	0.10 ± 0.00	0.05 ± 0.00
17:1	ND	0.03 ± 0.00
18:0	4.24 ± 0.00	3.30 ± 0.04
18:1n-9	38.3 ± 0.15	17.0 ± 0.33
18:2n-6	9.57 ± 0.04	14.5 ± 0.33
18:3n-3 *cis*	0.18 ± 0.00	58.0 ± 1.17
20:0	0.35 ± 0.00	0.10 ± 0.00
20:1n9	0.11 ± 0.00	0.09 ± 0.00
Total Saturated	51.0 ± 0.00	8.66 ± 0.00
Total Monounsaturated	38.6 ± 0.00	17.2 ± 0.00
Total n-6 Polyunsaturated	9.57 ± 0.00	14.5 ± 0.33
Total n-3 Polyunsaturated	0.18 ± 0.00	58.0 ± 1.17
Total Polyunsaturated	9.75 ± 0.00	72.5 ± 1.50

ND = not detected.

**Table 3:** Effect of 8 h postruminal infusion of propionate compared to acetate (SCFA) with and without palm oil on milk yield, plasma-free fatty acid profile molar proportion at h 8, and non-esterified fatty acid (NEFA) and glucose concentrations at hour 8 (after the last infusion dose) in Experiment 1. Means were determined to be different when *p* < 0.05 and tended to be different when 0.05 ≤ *p* ≤ 0.10.

Metabolite	Least Squared Means						*p*-Values		
	Ac ^1^	Palm-Ac ^2^	Palm-Pr ^3^	Pr ^4^	SEM	Trt	Palm Oil	SCFA	Palm Oil × SCFA
Milk Yield, kg/d	36.4	35.7	33.8	35.9	1.06	0.39	0.53	0.30	0.28
Baseline NEFA, mM	0.18	0.20	0.17	0.27	0.05	0.43	0.38	0.46	0.23
NEFA at h 8, mM	0.16	0.17	0.17	0.19	0.02	0.67	0.97	0.48	0.30
Baseline Glucose, mg/dL	48.3	49.5	44.6	47.3	2.60	0.63	0.79	0.29	0.48
Glucose at h 8, mg/dL	45.5	45.2	44.8	45.6	1.20	0.96	0.64	0.87	0.84
Plasma Fatty Acid, % mol	Ac ^1^	Palm-Ac ^2^	Palm-Pr ^3^	Pr ^4^	SEM	Trt	Palm oil	SCFA	Palm oil × SCFA
14:0	2.24	2.11	2.19	2.28	0.26	0.97	0.68	0.81	0.93
14:1	0.12	0.10	0.17	0.10	0.07	0.88	0.74	0.74	0.53
16:0	29.3	29.8	31.0	30.9	1.32	0.76	0.82	0.32	0.87
16:1	1.32	1.22	1.27	1.13	0.25	0.96	0.93	0.80	0.64
18:0	24.2	25.01	25.4	27.0	1.26	0.51	0.75	0.25	0.37
18:1n-9	21.8	23.3	23.3	21.1	2.38	0.89	0.47	0.89	0.88
18:2	16.3	14.3	13.3	15.6	1.10	0.27	0.34	0.12	0.44
18:3n-3 *cis*	1.92	1.61	1.54	1.63	0.17	0.43	0.26	0.30	0.52
20:4	2.29	2.16	1.51	1.96	0.38	0.52	0.46	0.23	0.68
20:5	0.35	0.31	0.25	0.29	0.03	0.15	0.15	0.07	1.0
22:6	0.08	0.07	0.07	0.07	0.01	0.70	0.91	0.42	0.42

^1^ Acetate postruminal infusion. ^2^ Palm oil and acetate postruminal infusion. ^3^ Palm oil and propionate postruminal infusion. ^4^ Propionate postruminal infusion.

**Table 4:** Effect of 8 h postruminal infusion of propionate compared to acetate (SCFA) with and without flaxseed oil on milk yield, plasma-free fatty acid profile molar proportion, and non-esterified fatty acid (NEFA) and glucose concentrations at hour 8 (after the last infusion dose) in Experiment 2. Means were determined to be different when *p* < 0.05 and tended to be different when 0.05 ≤ *p* ≤ 0.10.

Metabolite	LS Means						*p*-Values		
	Ac 2 ^1^	Flax-Ac ^2^	Flax-Pr ^3^	Pr 2 ^4^	SEM	Trt	Flax oil	SCFA	Flax Oil × SCFA
Milk Yield, kg/d	35.3	33.3	35.7	36.0	0.68	0.10	0.14	0.06	0.25
Baseline NEFA, mM	0.16	0.16	0.20	0.19	0.04	0.85	0.90	0.39	0.95
NEFA at h 8, mM	0.13	0.14	0.17	0.13	0.01	0.19	0.06	0.48	0.41
Baseline Glucose, mg/dL	50.5	50.6	53.1	50.8	2.0	0.77	0.56	0.48	0.58
Glucose at h 8, mg/dL	46.6	45.0	48.2	46.9	1.35	0.40	0.91	0.19	0.28
Plasma Fatty Acid, % mol	Ac 2 ^1^	Flax-Ac ^2^	Flax-Pr ^3^	Pr 2 ^4^	SEM	Trt	Flax oil	SCFA	Flax oil × SCFA
14:0	2.86 ^a^	1.91 ^b^	2.20 ^b^	2.84 ^a^	0.33	0.18	0.04	0.70	0.65
14:1	0.10	0.07	0.07	0.08	0.01	0.20	0.07	0.55	0.33
16:0	28.7 ^a^	24.7 ^b^	24.7 ^b^	29.4 ^a^	0.9	0.007	0.0009	0.76	0.71
16:1	1.08	0.89	0.94	0.88	0.07	0.23	0.37	0.31	0.11
18:0	27.1	25.7	24.5	28.9	1.42	0.22	0.07	0.80	0.32
18:1	22.2	21.62	22.1	19.9	1.20	0.54	0.51	0.50	0.28
18:2	13.5	15.0	15.0	13.5	0.85	0.43	0.11	0.97	0.99
18:3n-3 *cis*	1.59 ^b^	7.34 ^a^	8.00 ^a^	1.57 ^b^	0.77	0.0002	0.0001	0.69	0.67
20:4	2.44	2.40	2.11	2.47	0.28	0.79	0.50	0.65	0.58
20:5	0.33	0.32	0.30	0.35	0.03	0.74	0.34	1.0	0.60
22:6	0.09	0.07	0.07	0.08	0.02	0.81	0.39	0.74	0.84

^1^ Acetate postruminal infusion. ^2^ Flaxseed oil and acetate postruminal infusion. ^3^ Flaxseed oil and propionate postruminal infusion. ^4^ Propionate postruminal infusion. ^a,b^ denote significant treatment differences as determined by *p* < 0.05.

**Table 5:** Effect of postruminal infusion of propionate or acetate (SCFA) with and without palm oil (Experiment 1) or the infusion of propionate or acetate (SCFA) with and without flaxseed oil (Experiment 2) on the mRNA abundance of hepatic pyruvate carboxylase (*PC*) and phosphoenolpyruvate carboxykinase isoform 1 (*PCK1*) and their ratios.

mRNA Abundance ^1^	Means					*p*-Values			
Experiment 1	Ac ^2^	Palm-Ac ^3^	Palm-Pr ^4^	Pr ^5^	SEM	Trt	Palm Oil	SCFA	Palm Oil × SCFA
*PC*	0.89	2.05	2.34	2.88	1.63	0.80	0.84	0.46	0.58
*PCK1*	4.90	3.64	1.06	4.14	2.0	0.60	0.30	0.43	0.68
*PC:PCK1*	0.98	1.02	0.31	0.84	0.47	0.74	0.64	0.40	0.57
Experiment 2	Ac 2 ^6^	Flax-Ac ^7^	Flax-Pr ^8^	Pr 2 ^9^	SEM	Trt	Flax Oil	SCFA	Flax oil × SCFA
*PC*	0.36	0.39	0.34	0.32	0.06	0.84	0.63	0.47	0.97
*PCK1*	1.22	0.85	1.0	0.82	0.29	0.76	0.77	0.68	0.37
*PC:PCK1*	0.34	0.47	0.95	0.39	0.34	0.59	0.34	0.46	0.54

^1^ Arbitrary units. ^2^ Acetate postruminal infusion for Experiment 1. ^3^ Palm oil and acetate postruminal infusion for Experiment 1. ^4^ Palm oil and propionate postruminal infusion for Experiment 1. ^5^ Propionate postruminal infusion for Experiment 1. ^6^ Acetate postruminal infusion for Experiment 2. ^7^ Flaxseed oil and acetate postruminal infusion for Experiment 2. ^8^ Flaxseed oil and propionate postruminal infusion for Experiment 2. ^9^ Propionate postruminal infusion for Experiment 2.

## Data Availability

All data will be made available upon request to the corresponding author.
